# Contrasting responses of two *Camellia oleifera* rootstocks differing in phosphorus efficiency to low-phosphorus stress

**DOI:** 10.1186/s12870-026-09197-z

**Published:** 2026-06-15

**Authors:** Xuyi Tang, Wenjuan Su, Tingting Zou, Yang Liu, Haolin Zhang, Dongnan Hu, Juan Liu

**Affiliations:** 1 Jiangxi Provincial Key Laboratory of Subtropical Forest Resources Silviculture, Jiangxi Agricultural University, Nanchang, 330045 China; 2 State Key Laboratory of Tree Genetics and Breeding, Nanjing Forestry University, Nanjing, 210037 China

**Keywords:** Low P stress, Gene expression profile, Regulatory mechanisms, Rootstocks, Breeding

## Abstract

**Background:**

*Camellia oleifera* Abel, the dominant woody oil crop of southern China, often suffers from limited productivity due to the prevalence of phosphorus (P) stress in acidic soils. While grafting is an effective strategy to mitigate nutrient scarcity, the mechanistic basis of rootstock-mediated P tolerance in this species remains poorly understood. This study investigated the morphological, physiological, and molecular responses of two contrasting *C. oleifera* rootstocks, the P-tolerant Ganwu 1 (GW1) and the P-sensitive Ganwu 2 (GW2) under P-deficient conditions.

**Results:**

Our findings identified rootstock types as the primary determinant of P tolerance. GW1 exhibited superior resilience by re-engineering its root architecture, achieving a 4.12-fold higher root biomass and significantly greater 66.5% increase in fine root volume than GW2 under low P stress. In contrast, GW2 showed a compensatory but inefficient root expansion that failed to enhance soil exploration. Specifically, GW1 demonstrated a 3.7-fold higher root P accumulation and a 52.4% increase in root P-utilization efficiency under stress, reflecting a significantly higher capacity for P acquisition and internal allocation. This was coupled with enhanced nutrient rebalancing, particularly through robust potassium (K) accumulation in shoots, whereas GW2 suffered from a significant decline in soluble protein content and higher lipid peroxidation (MDA). Additionally, GW1 sustained high root acid phosphatase (ACP) activity during late-stage stress to further mobilize P. At the molecular level, GW1 was characterized by the accelerated induction of the *CoARF22*, *CoPHR1*, and *CoPHT1;1* and earlier activation (70 days) of *CoALMT*-mediated organic acid genes. These proactive responses enabled GW1 to effectively mobilize soil P and maintain structural integrity, establishing it as a highly efficient rootstock for P-limited acid soils.

**Conclusions:**

This integrated "morphology-physiology-signaling" framework grants GW1 a superior low-P adaptive advantage. These results identify GW1 as a promising, P-efficient rootstock for sustainable *C. oleifera* production in P-deficient environments.

## Background

Phosphorus (P), an essential element for plants, plays pivotal roles in cellular metabolism, energy transduction, and stress resistance. Despite its relative abundance in soils, P fixation renders it largely unavailable, creating a major constraint for global agricultural productivity [1]. While P fertilizers are commonly applied, plant utilization efficiency remains low (15 ~ 25%) [2], with excess accumulation causing serious environmental concerns. Given the gradual depletion of P rock reserves, developing P-efficient cultivars has emerged as a critical strategy for sustainable agriculture [3–5].

Extensive research has elucidated the sophisticated adaptive mechanisms plants employ to cope with P deficiency, which can be broadly categorized into morphological, biochemical, and molecular responses. At the morphological level, plants optimize their root system architecture in response to low P availability by increasing lateral root formation, elongating root hairs, and enhancing root branching angles to explore larger soil volumes [6, 7]. Biochemically, plants exudate acid phosphatases to mineralize labile organic P and release organic acids to solubilize fixed inorganic P [8]. Simultaneously, they upregulate antioxidant enzymes, such as peroxidases, to counteract oxidative stress induced by P deficiency [9]. Extensive molecular studies have identified a complex regulatory network governing plant P nutrition, involving coordinated interactions among multiple biological components, including high-affinity phosphate transporters (*PHTs*), P starvation-responsive transcription factors (*PHRs*,* WRKYs*), root exudation (e.g. aluminum-activated malate transporters, *ALMT*) and phytohormone signaling (e.g. auxin response factors, *ARF*) [5, 10, 11].

Notably, significant interspecies and intraspecies variations exist in P efficiency. For instance, P-efficient apple cultivars demonstrate enhanced root proliferation and rhizosphere acidification [9], while cotton genotypes exhibit differential peroxidase activity under low P conditions [12]. White lupin (*Lupinus albus* L.) develops specialized cluster roots that efficiently mobilize soil P through localized exudation of carboxylates and phosphatases [13]. These diverse adaptation strategies are underpinned by the differential expression of P-responsive genes that fine-tune P homeostasis. Understanding the genetic and physiological basis of these variations offers valuable targets for breeding P-efficient crop varieties and selecting resilient rootstock-scion combinations.

*Camellia oleifera* Abel, a premium woody oil crop indigenous to southern China, encounters substantial growth restrictions in the P-deficient red soils predominant in its cultivation areas [14–16]. These highly weathered ultisols and oxisols exhibit a remarkable P-fixation capacity, with typically less than 5% of total P remaining in available forms [17]. This severe P limitation is intrinsically linked to the inherent soil acidity, which facilitates the activation of iron (Fe) and aluminum (Al) ions that rapidly bind with inorganic P to form insoluble complexes. Consequently, this pH-dependent P-fixation creates profound constraints on the growth, development, and oil production potential of *C. oleifera* [16].

Nurse-seed grafting with elite scion cultivars has become the predominant propagation method for *C. oleifera*, contributing to significant yield enhancement and widespread adoption in commercial plantations [18, 19]. Extensive horticultural researches in other fruit crops have demonstrated the effectiveness of grafting techniques in addressing cultivation limitations, particularly through rootstock-mediated improvements in soil resource acquisition and maintenance of superior fruit quality traits from scion cultivars [20]. Previous studies have explicitly examined P-use divergence among cultivars. For example, the vigorous variety of the “Gan 8” scion showed better performance in shoot P accumulation [19]. In commercial cultivation, the selection of *C. oleifera* rootstocks for grafting is typically based on the use of conspecific seeds (same-species) to ensure high graft compatibility, yet there remains a limited mechanistic understanding of rootstock-specific effects on P acquisition and utilization efficiency. Our preliminary investigation systematically evaluated ten commonly used rootstock types through comprehensive P efficiency screening, assessing biomass accumulation, root morphological traits, tissue P concentration, and P use efficiency (unpublished). According to principal component analysis (PCA) and the membership function method, this screening identified distinct P tolerance phenotypes: “Ganwu 1” (named GW1, P-efficient type) and “Ganwu 2 (named GW2, P-sensitive type). However, the physiological adaptations and molecular regulation underlying these contrasting P efficiency responses remain unexplored.

In this study, we investigated the divergent responses of two *C. oleifera* rootstocks to low P stress, focusing on whether the superior P-acquisition of the tolerant genotype (GW1) is primarily driven by the coordinated adjustment of root morphological traits, specifically the proliferation of fine-root classes rather than a simple expansion of total biomass. We also examined how these morphological shifts align with nutrient uptake efficiency and the expression of key P-responsive genes. This approach allows us to characterize the distinct strategies of different rootstocks by integrating root system composition with molecular and nutrient-accumulation data, providing a clearer understanding of the traits that contribute to P-efficiency in acidic red soils.

## Methods

### Plant materials

The plant materials used in this study consisted of two distinct half-sib families of *C. oleifera* as rootstocks, derived from open-pollinated seeds of two maternal genotypes, GW1 and GW2. These rootstock seedlings were bud-grafted with a common elite scion cultivar, “Changlin 4” (named CL4), which is a variety widely cultivated in the primary production regions of China. This experimental design allowed for the evaluation of the differential influence of rootstock genetic background on P resilience while maintaining a consistent scion genotype. The experiment was conducted at Jiangxi Agricultural University (115°92′E, 28°68′N) in a subtropical monsoon climate zone characterized by mean annual temperature of 17.5 °C, annual precipitation of 1, 596.4 mm. The site’s relatively flat terrain and optimal solar exposure provided ideal conditions for pot-based seedling cultivation trials.

### Experiment design

Mature fruits of *C. oleifera* (GW1 and GW2 genotypes) were collected in 2020, sun-dried, and manually processed to obtain seeds, which were then stratified in sand during December 2020. Following germination in May 2021, uniform seedlings were selected as rootstocks and grafted with semi-lignified scions of “CL4” using the cleft grafting method [18, 19]. After approximately one year, the successfully grafted seedlings were transplanted into 18-cm diameter pots, which were filled with a 1:3 (v/v) mixture of sand and red soil collected from the planting area. Initial soil chemical properties were determined according to the procedure in previous literature [21]. Briefly, available P (12.20 ± 0.89 mg kg^− 1^) was measured using the Bray I method; inorganic N (56.99 ± 11.32 mg kg^− 1^) was determined via the alkali-hydrolytic diffusion method; and available K (3.45 ± 0.37 mg kg^− 1^) was measured by flame photometry. The plants were maintained in a greenhouse. Standard horticultural practice was maintained by applying 100 mL of tap water weekly. The irrigation volume was dynamically adjusted according to weather conditions, increasing irrigation during periods of high temperature and reducing it following rainfall. Our previous trials have demonstrated that *C. oleifera* can persist in P-deficient red soils; consequently, 0 mM P has been established and repeatedly used in our published studies as the threshold for P-stress experiments [15, 19, 22]. Two P regimes were therefore imposed: P0 (0 mmol L^− 1^, low P stress) and P1 (1 mmol L^− 1^, control). Different concentrations of KH_2_PO_4_ as the P source were applied with each irrigation of Hoagland’s nutrient solution, and KCl was added to maintain ionic balance. Each plant was irrigated with approximately 100 ml of 1/2 Hoagland nutrient solution every month. Thirty replicates per treatment were set. Whole plants of both P-efficient (GW1) and P-sensitive (GW2) rootstocks were collected and harvested for analysis at four critical growth stages under P-deficient and sufficient conditions, including pretreatment baseline (Day 0), spring shoot sprouting (Day 70), summer shoot sprouting (Day 140), and autumn shoot sprouting (Day 210).

### Plant growth and nutrient measurements

Height and basal diameter were measured every 70 days. Incremental growth was defined as the cumulative increase in height or diameter at each sampling time point relative to the initial measurement (day 0). The stem, leaves and root systems were separately harvested. Some samples were stored at -80 °C freezer for physiological analysis and transcription analysis. The remaining samples were oven-dried at 72 °C for 48 h to reach a constant weight, and their respective dry biomass was recorded. Total shoot biomass was calculated as the sum of stem and leaf dry weights, which was then used to determine the root-to-shoot ratio.

The dried samples were ground into a fine powder using a ball mill and passed through a 0.5-mm sieve to ensure homogeneity for subsequent elemental analysis. Following the H_2_SO_4_-H_2_O_2_ digestion method described by Li et al. [23], total N element concentrations (*mg g*^*− 1*^) were determined using an automatic discrete chemical analyzer (Smartchem 200, AMS, Italy). Total K element concentrations (*mg g*^*− 1*^) were measured using a flame photometer (Yuanxi F-100, Shanghai Yuanxi, China). Total P element concentrations (*mg g*^*− 1*^) were quantified using the molybdenum antimony anti-colorimetric method [23]. P accumulation and utilization efficiency for the whole plant (including roots, stems, and leaves) were calculated based on the following equations according to Li et al. [23].

1 $$\:\mathrm{P}\:\mathrm{A}\mathrm{c}\mathrm{c}\mathrm{u}\mathrm{m}\mathrm{u}\mathrm{l}\mathrm{a}\mathrm{t}\mathrm{i}\mathrm{o}\mathrm{n}\:\left(\mathrm{m}\mathrm{g}\right)=\mathrm{T}\mathrm{o}\mathrm{t}\mathrm{a}\mathrm{l}\:\mathrm{P}\:\mathrm{c}\mathrm{o}\mathrm{n}\mathrm{c}\mathrm{e}\mathrm{n}\mathrm{t}\mathrm{r}\mathrm{a}\mathrm{t}\mathrm{i}\mathrm{o}\mathrm{n}\mathrm{s}\:\times\:\:\mathrm{D}\mathrm{r}\mathrm{y}\:\mathrm{b}\mathrm{i}\mathrm{o}\mathrm{m}\mathrm{a}\mathrm{s}\mathrm{s}\:\:$$

2 $$\:\mathrm{P}\:\mathrm{U}\mathrm{t}\mathrm{i}\mathrm{l}\mathrm{i}\mathrm{z}\mathrm{a}\mathrm{t}\mathrm{i}\mathrm{o}\mathrm{n}\:\mathrm{E}\mathrm{f}\mathrm{f}\mathrm{i}\mathrm{c}\mathrm{i}\mathrm{e}\mathrm{n}\mathrm{c}\mathrm{y}\:(\mathrm{P}\mathrm{U}\mathrm{E},\:\mathrm{g}\:{\mathrm{g}}^{-1})\:=\frac{Total\:dry\:biomass\:}{P\:accumulation}$$

### Root morphology analysis

Following thorough rinsing with deionized water, complete root systems were carefully arranged in a transparent acrylic imaging tray and scanned using an Expression 10000XL 3.49 high-resolution root scanner (Epson Telford Ltd., UK). The resulting digital images were analyzed with WinRHIZO Pro 2012b software (Regent Instruments Inc., Quebec, Canada) to quantify three key architectural parameters: (1) total root length (cm), (2) root surface area (cm²), (3) root volume (cm³), and (4) root projection area (cm^2^). The roots were further categorized based on root diameter into fine roots (0–2 mm), small-diameter roots (2–4 mm), and thick roots (≥ 4 mm).

### Root physiology analysis

Malondialdehyde (MDA) content was determined using the thiobarbituric acid (TBA) method with absorbance measurements at 532 nm (primary peak), 600 nm (turbidity correction), and 450 nm (background correction). Soluble protein content (SPC) was quantified via the Bradford method using Coomassie Brilliant Blue G-250 with bovine serum albumin (BSA) as standard (A₅₉₅ nm).

Enzyme activities were assayed using Nanjing Jiancheng Bioengineering Institute kits, including peroxidase (POD, A084-3-1), catalase (CAT, A007-1-1; ammonium molybdate method), superoxide dismutase (SOD, A001-1-2; hydroxylamine method), and acid phosphatase (ACP, A060-1-1; spectrophotometric method). Root tissues were homogenized to determine the total activities of these enzymes. POD and SOD activities were expressed per gram of fresh weight (*U g*^*− 1*^ FW) to ensure data stability across different tissue types. CAT and total ACP activities were normalized to the total soluble protein content of the root extracts and expressed as *U mg*^*− 1*^ prot. All enzymatic assays were performed strictly following the manufacturer’s protocols to ensure reproducibility.

### RNA extraction, cDNA synthesis and gene expression by qRT-PCR

Total RNA was extracted from *C. oleifera* roots stored at − 80 °C using a specialized kit for polysaccharide-rich samples (DP441, Tiangen, China), with integrity verified by 1.0% agarose gel electrophoresis. High-quality total RNA with 1 µg was used per sample followed by cDNA synthesis using iScript™ kit (BIO-RAD). Based on established P signaling models in *C. oleifera* and related woody species [15, 24–26], five core P-responsive genes were selected for molecular analysis. These include the central signaling regulators *CoPHR1*,* CoARF22*, and *CoWRKY53*, alongside the downstream functional genes *CoALMT* (associated with organic acid exudation) and *CoPHT1;1* (involved in P uptake). This selection strategy ensures a holistic assessment of the transcriptional shifts across different levels of the P-starvation response pathway. Primers were designed using Primer3Plus and listed in Table 1. *CoEF1α* was chosen as the internal reference gene, given its validated stability and widespread application in numerous *C. oleifera* studies [10, 15, 22]. The qRT-PCR reaction (25 µL) contained 9.5 µL ddH₂O, 1 µL each primer, 1 µL cDNA and 12.5 µL MasterMix, using the following program: 94 °C/3min; 30 cycles of 94 °C/30s, 55 °C/30s, 72 °C/1min; 72 °C/5min. qRT-PCR was performed in 20 µL reactions containing 10 µL SYBR Green Supermix, 1 µL each primer (10 µM), 1 µL cDNA and 7 µL ddH₂O, with cycling conditions: 95 °C/3min; 40 cycles of 95 °C/10s, 60 °C/30s. For each treatment group (including GW1 and GW2 rootstocks under low P stress at different time points), three independent plants were randomly selected as biological replicates. Each biological replicate sample was subjected to three parallel qRT-PCR reactions as technical replicates. All qRT-PCR reactions were subjected to the 2^−ΔΔCt^ method for relative quantification [27], with subsequent data normalization against validated reference genes and statistical analysis performed across biological replicates.

**Table 1:** Primer information for qRT-PCR

Gene	description	length (bp)	Primers Sequence (5’-3’)
*CoEF1a1*	Reference gene	160	*F*:CAAAGAAGGGTGCCAAGTGA
			*R*:ACCAAACAACCGACCTACGA
*CoPHR1*	A transcription factor regulating P starvation-responsive	200	*F*: ACCAATCTGCCTGCGGA
			*R*: CCCAAACGATACTTCTG
*CoARF22*	Involved in auxin signaling transduction and root development	180	*F*: TGCTAACAATGGTGGAGGGT
			*R*: CACCGTGAACATCCTTTGCA
*CoWRKY53*	A transcription factor that responds to both biotic and abiotic stresses	210	*F*: CACCCATCACCAATTTGCCA
			*R*: AGCAAACCCTCACTTGTCCT
*CoALMT*	An activated transporter that maintains ion homeostasis	195	*F*: GTTTGGGCAATCCTCACTGT
			*R*: CTGTCATCTTCCCAGCCATT
*CoPHT1;1*	Mediates the uptake of inorganic P from soil or mycorrhizal networks	192	*F*: GTGGCTCGGTGACAAGATGGG
			*R*:CATAATGGTAGCAGAAAGAGGGT

### Statistical analysis

Statistical analyses were performed using IBM SPSS Statistics 22 (IBM Corp., Armonk, NY, USA). To evaluate the effects of rootstocks, P treatments, and growth duration, a three-way analysis of variance (ANOVA) was conducted. The main effects of rootstocks (R), P treatment (T), and day (D), as well as their interactions (*R × T*,* R × D*,* T × D*, and *R × T × D*), were explicitly tested. Tukey’s HSD (Honestly Significant Difference) test was used for post-hoc multiple comparisons among treatments at a significance level of *P* ≤ 0.05. Principal component analysis (PCA) was conducted on Z-score standardized data to visualize overall physiological variations. Graphical representations were constructed using Origin 2021. In this study, ** and * indicate significance at *P* ≤ 0.01 and *P* ≤ 0.05, respectively.

## Results

### Growth increment of two rootstocks under P limited

**Fig. 1:**
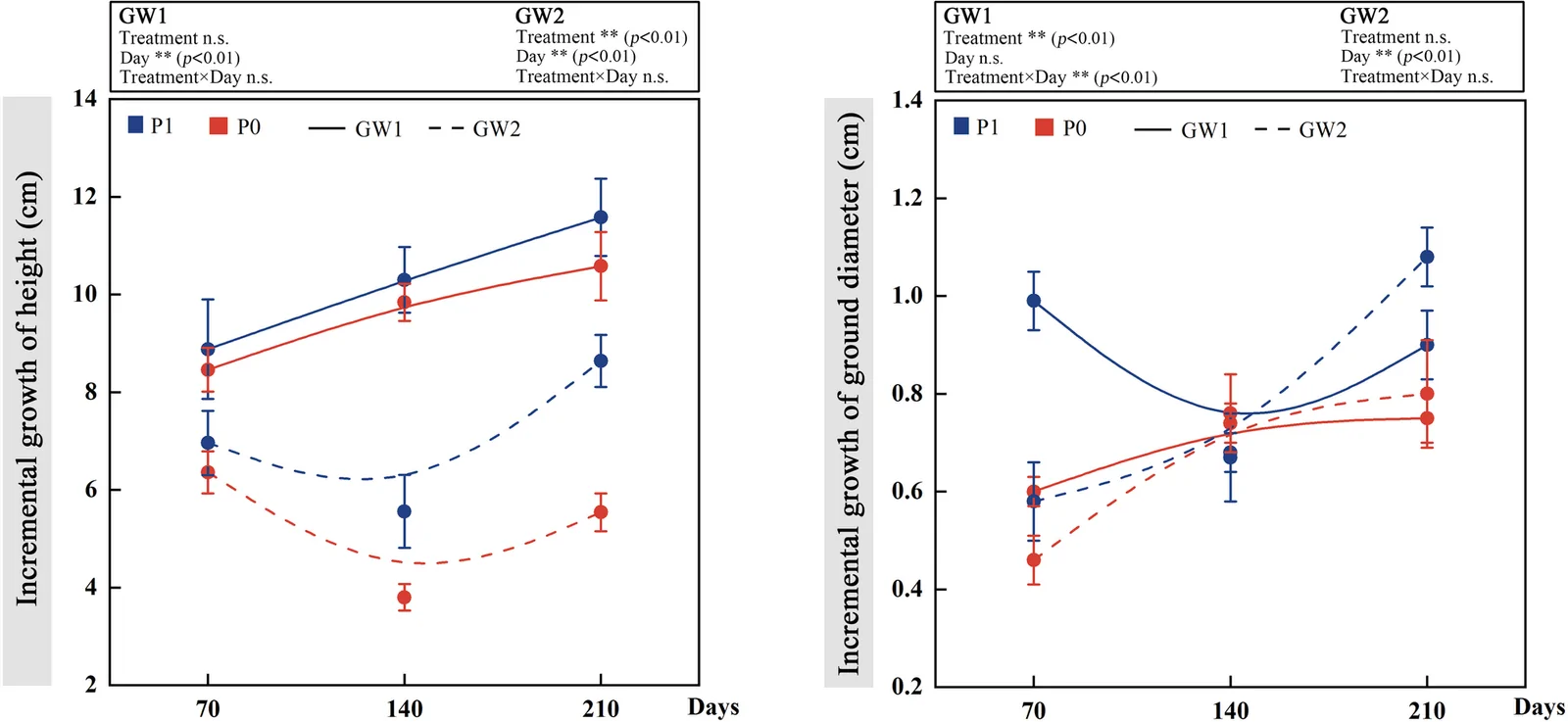
Growth increments in plant height and ground diameter of two *C. oleifera* rootstocks (GW1 and GW2) under P0 and P1, observed at 70, 140, and 210 days. In the figure legend, “Treatment”, “Day”, and “Day*×*Rootstocks” represented the effects of P level, time, and their interaction, respectively. ** indicated significant effect at the *P* ≤ 0.01 level; absence of notation indicates no significant difference in the interaction effect (*N* = 5). Data are presented as mean ± SE

low P stress significantly suppressed the incremental growth of *C. oleifera* (Fig. 1), with marked genotypic disparities between the P-efficient (GW1) and P-sensitive (GW2) rootstocks. Under low-P conditions (P0), both rootstocks exhibited reduced height increment compared to P-sufficient controls (P1). Notably, GW1 showed greater growth performance than GW2 during advanced developmental stages. Under 210 days of P starvation, seedling height was reduced by 25.93% in GW2 and 16.67% in GW1 compared to their respective controls. Regarding basal diameter, GW2 exhibited significant variations across P gradients, whereas no significant differences were observed in GW1 across varying P availabilities throughout the study period.

**Fig. 2:**
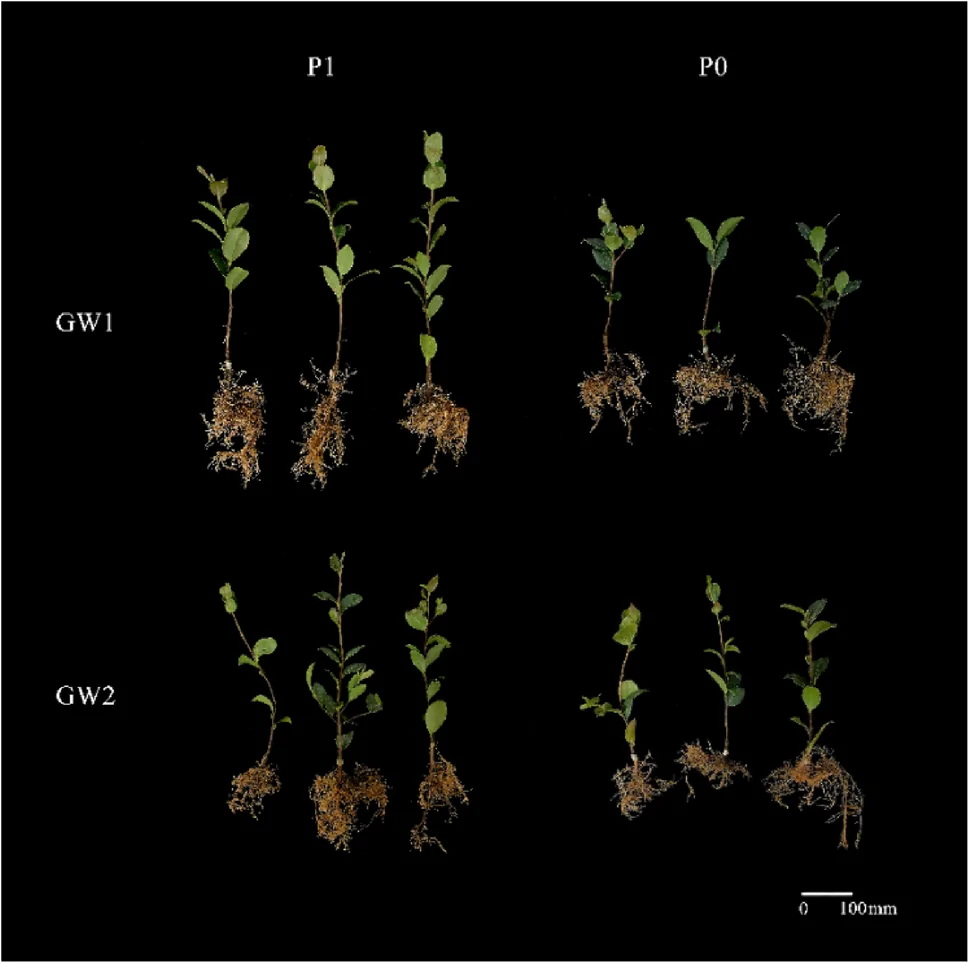
Root morphological comparison of *C. oleifera* grafted seedlings at 210 days under P0 and P1

Root system analysis revealed distinct morphological adaptation strategies between rootstock types under P limitation (Fig. 2 & Table 2). The P-efficient GW1 rootstock exhibited remarkable plasticity, characterized by a 66.5% increase in fine root volume compared to sufficient P conditions (P1). This adaptation corresponded with significant elongation of fine roots, indicative of superior P acquisition capacity. In contrast, the P-sensitive GW2 demonstrated severe growth repression under P0 treatment, with total root length decreasing by 58.4–82.1% across all root orders. In GW2, the observed increase in root surface area was not reflected in the root projection area, suggesting a divergence in morphological efficiency. This is attributed to the proliferation of fine roots with minimal diameters. While these Small-diameter and thick roots significantly elevate total surface area, their fine roots projection area remains low due to their slender geometry and tendency to cluster. Consequently, the increased surface area in GW2 represents a redundant structural investment that fails to enhance spatial soil exploration, whereas the root architecture of GW1 more effectively balances surface area with projection area to maximize P scavenging.

**Table 2:** Determination of root system morphology

Grafting combination	GW1 + CL4		GW2 + CL4	
Treatment	P0	P1	P0	P1
fine root length/cm	1443.34 ± 23.69a	1156.94 ± 68.51b	516.89 ± 6.12c	1437.88 ± 5.54a
Small-diameter root length/cm	10.66 ± 1.08b	12.23 ± 1.10ab	2.811 ± 0.50c	15.01 ± 0.37a
thick root length/cm	8.82 ± 4.48a	8.39 ± 2.50a	3.60 ± 0.53b	8.66 ± 0.51a
fine root volume/cm^3^	3.33 ± 0.17a	2.00 ± 0.18b	0.97 ± 0.07c	2.87 ± 0.18a
Small-diameter root volume/cm^3^	0.69 ± 0.07b	0.70 ± 0.05b	1.17 ± 0.13a	0.94 ± 0.04ab
thick root volume/cm^3^	2.853 ± 0.05a	2.26 ± 0.07c	2.09 ± 0.18c	2.49 ± 0.12ab
fine root surface area/cm^2^	215.49 ± 15.89a	147.24 ± 11.33b	70.41 ± 8.6c	193.41 ± 8.22ab
Small-diameter root surface area/cm^2^	9.39 ± 0.78b	10.19 ± 0.45b	37.54 ± 1.80a	13.39 ± 2.26b
thick roots surface area/cm^2^	17.31 ± 0.66b	15.09 ± 0.52b	39.70 ± 0.82a	16.07 ± 0.76b
fine root projection area/cm^2^	3.33 ± 0.17a	2.00 ± 0.08b	0.97 ± 0.07c	2.87 ± 0.23a
Small-diameter projection area/cm^2^	0.69 ± 0.07b	0.70 ± 0.09b	1.17 ± 0.03a	0.95 ± 0.07ab
Thick roots projection area/cm^2^	2.85 ± 0.14a	2.26 ± 0.14b	2.09 ± 0.03b	2.49 ± 0.05ab

Data are presented as mean ± SE. Different lowercase letters above the bars indicate significant differences between treatments and rootstock types based on Tukey’s HSD test (*P ≤ 0.05*)

### Biomass allocation of two rootstock types under P limitation

**Fig. 3:**
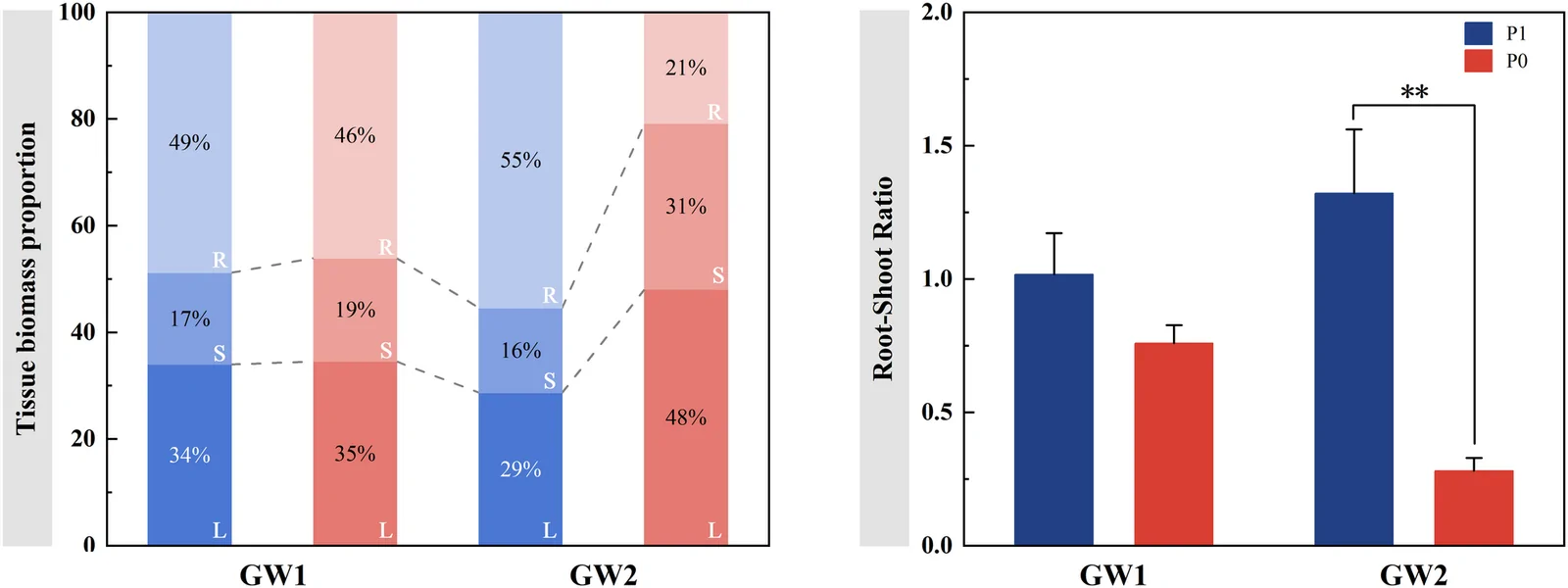
Biomass allocation and root-to-shoot ratios of two *C. oleifera* rootstocks under varying P supply. Seedlings were grown under P-deficient (P0) and P-sufficient (P1) conditions for 210 days. Left: Biomass distribution among roots (R), stems (S), and leaves (L). Right: Root-to-shoot ratios (where shoot represents the sum of stem and leaf biomass). Blue and red bars represent P1 and P0 treatments, respectively. Asterisks (**) denote significant differences at *P* ≤ 0.01; absence of notation indicates no significant difference (*N* = 3). Data are presented as mean ± SE

Under P-limiting conditions, grafted *C. oleifera* seedlings exhibited striking rootstock typic differences in biomass partitioning (Fig. 3, left). The P-efficient GW1 rootstock demonstrated superior adaptive plasticity, achieving a root dry weight 4.12-fold greater than that of GW2 under low P stress. Notably, GW1 maintained a balanced allocation strategy, with its stem biomass increasing by 41.7% relative to P-sufficient controls while preserving stable leaf production. In contrast, the P-sensitive GW2 rootstock experienced severe biomass suppression under low P stress. Its root system suffered a 78.6% reduction compared to normal P conditions, leading to a significant collapse in its root-to-shoot ratio (Fig. 3, right). These divergent responses underscore GW1’s capacity to prioritize root development to sustain overall plant vigor under chronic nutrient stress.

### Nutrient distribution of two rootstocks under P limitation

**Fig. 4:**
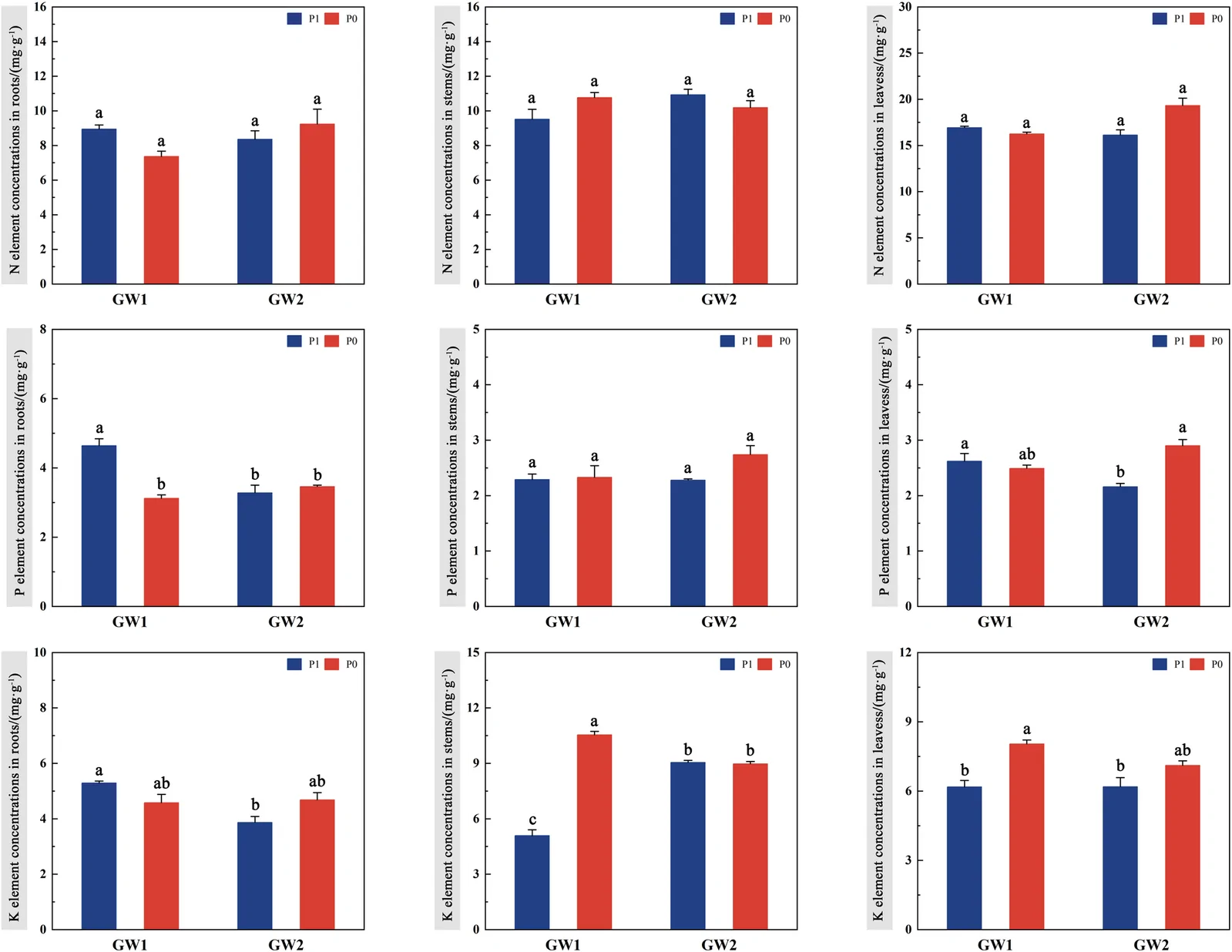
Effects of P availability on nutrient concentration and partitioning in *C. oleifera* rootstock combinations. Concentrations of N, P and K in different plant organs under P1 and P0 conditions. Different lowercase letters above the bars indicate significant differences between treatments and rootstock types based on Tukey’s HSD test (*P ≤ 0.05*). Values are presented as means ± SE (*N* = 3)

low P stress induced distinct nutrient allocation strategies between rootstock types (Fig. 4). Root P analysis revealed that GW1 demonstrated a significant 32.8% reduction in root P concentration, whereas GW2 accumulated 5.5% more root P. Notably, GW2 exhibited a significant 34.3% increase in leaf P concentration. In contrast, GW1 maintained foliar P stability, reflecting more efficient internal P reallocation. Furthermore, GW1 demonstrated superior K homeostasis under low P stress, exhibiting 2.07-fold and 1.30-fold increases in stem and leaf K element concentrations respectively compared to sufficient P conditions, while GW2 maintained a compromised K status with no significant differences between the two P treatments. This differential resource partitioning highlights GW1’s adaptive capacity for coordinated nutrient rebalancing under P scarcity.

**Fig. 5:**
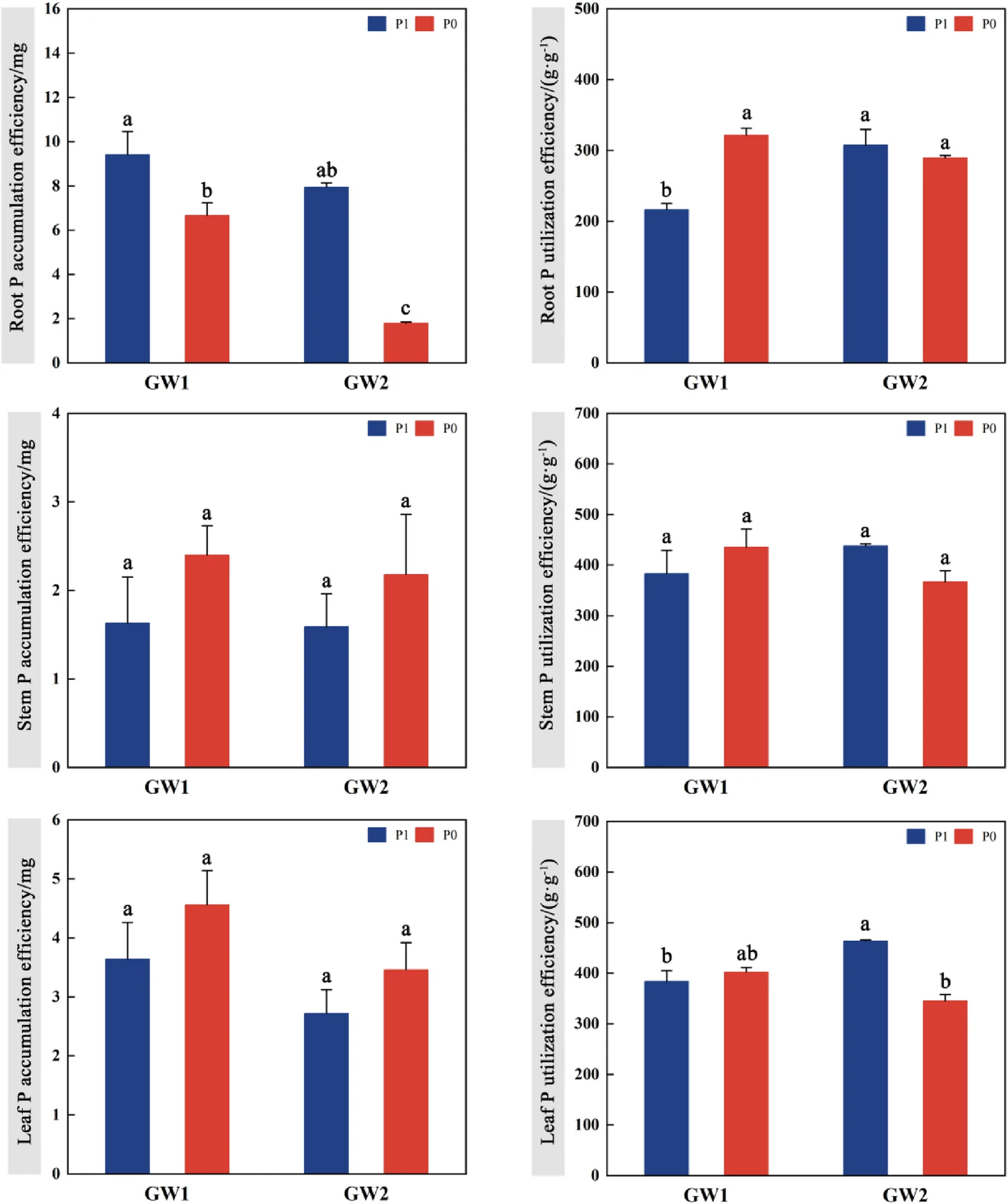
P accumulation and P utilization efficiency in roots, stems, and leaves of two *C. oleifera* rootstock (GW1 and GW2) under P0 and P) at 210 days. Different lowercase letters above the bars indicate significant differences between treatments and rootstock types based on Tukey’s HSD test (*P ≤ 0.05*). Values are presented as means ± SE (*N* = 3)

A comparative evaluation of P accumulation and utilization demonstrated that GW1 rootstocks exhibited significantly higher capacity for P acquisition and efficient internal allocation under low P stress (Fig. 5). Specifically, under P0 treatment, GW1 maintained a 3.7-fold higher root P accumulation compared to GW2, directly associating with its superior root foraging capacity. Regarding utilization, GW1 displayed systemic optimization in P remobilization, characterized by a significant 52.4% increase in root P utilization efficiency under stress. In contrast, while GW1 successfully maintained stable P utilization efficiency in leaves, GW2 suffered a significant reduction in leaf P utilization efficiency, dropping to levels markedly lower than its P-sufficient controls. This divergence highlights GW1’s superior adaptive capacity for coordinated nutrient acquisition and utilization under P scarcity.

### Physiological responses of two rootstocks under P limitation

**Fig. 6:**
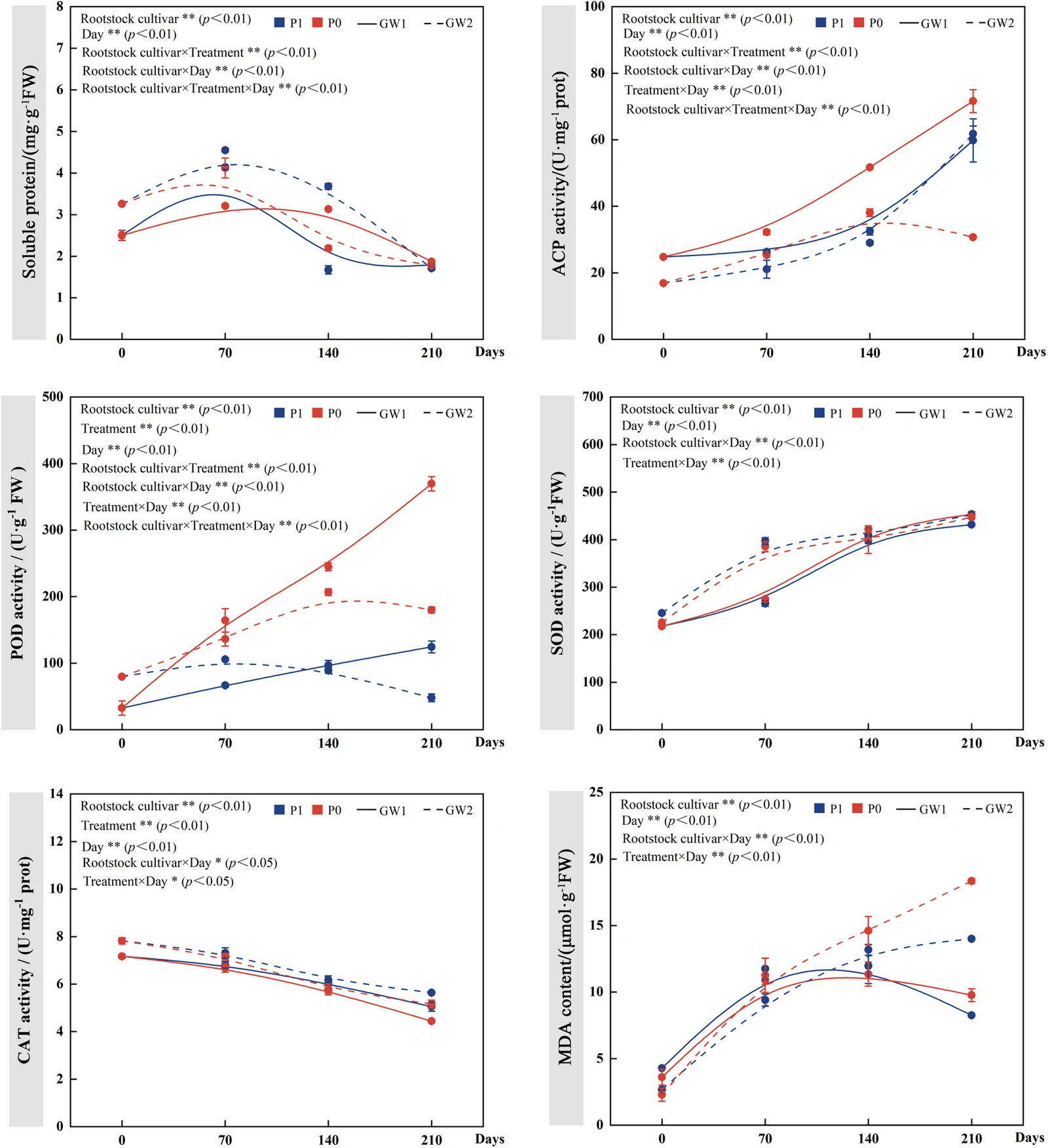
Differences in antioxidant enzyme activities and membrane lipid peroxidation products in the roots of two *C. oleifera* rootstocks (GW1 and GW2) under P0 and P1 at 0d, 70d, 140d and 210 d. * and ** indicated significant effects at the *P* ≤ 0.05 and *P* ≤ 0.01 levels, respectively; absence of notation indicated no significant difference in the interaction effect (*N* = 3). Data are presented as mean ± SE

Comparative analysis revealed distinct enzymatic and metabolic adaptation strategies between the two rootstocks (Fig. 6). Under low P stress, GW1 sustained significantly higher total root ACP activity and a 2–4 fold increase in POD activity during the late stress stage (140–210 d). In contrast, while GW2 showed an initial induction, its total ACP and POD responses were transient and declined sharply after 140 d. Furthermore, GW1 maintained stable soluble protein content and SOD activity, effectively mitigating oxidative damage as evidenced by its consistently low MDA levels. Conversely, GW2 suffered from a significant decline in protein content and a progressive accumulation of MDA, which peaked at 210 d. These results indicate that GW1 maintains superior cellular homeostasis and a more robust antioxidant defense system to withstand prolonged low P stress.

### PCA-based evaluation of rootstock responses to P limitation

**Fig. 7:**
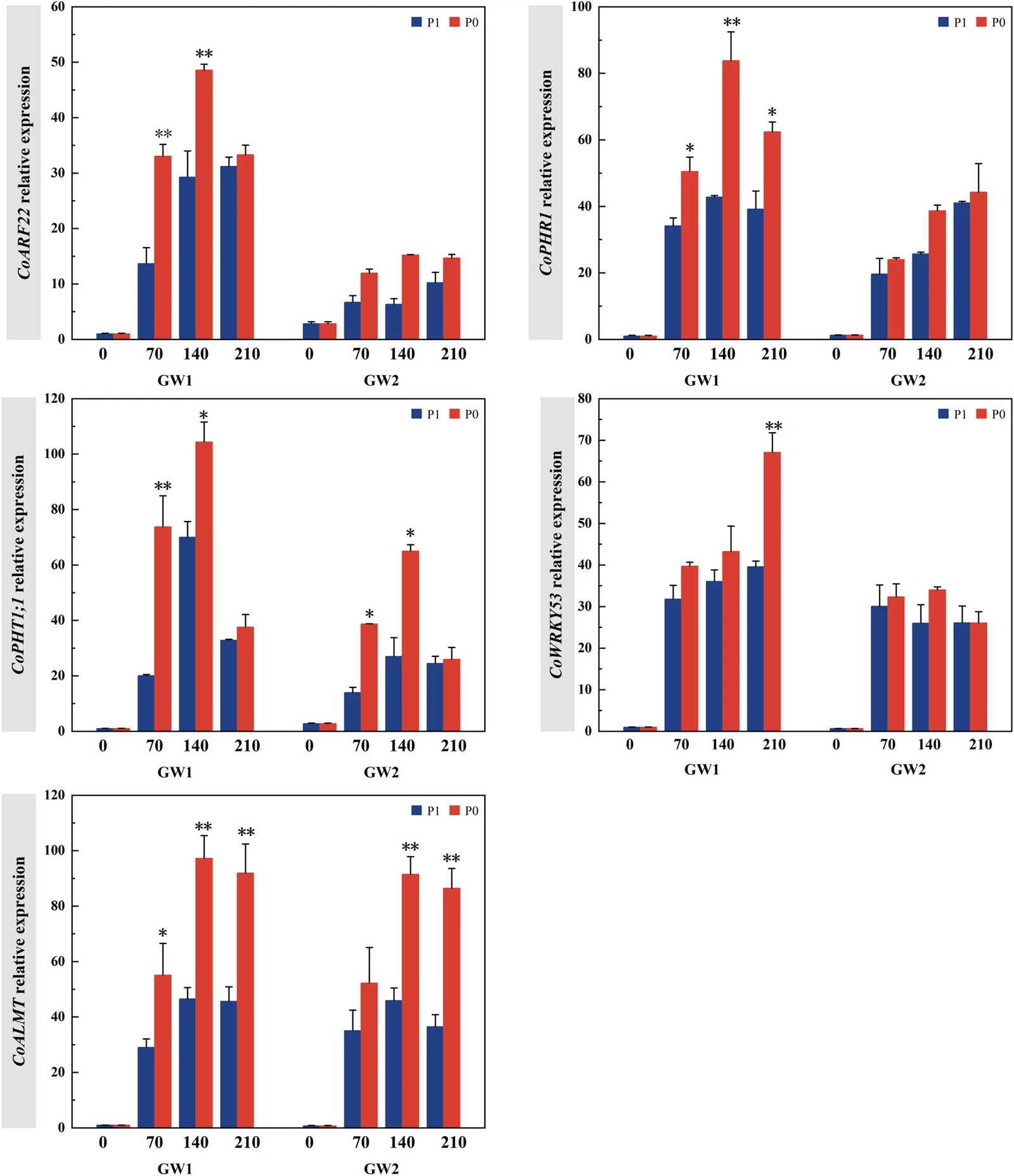
Principal component analysis of 23 traits for two *C. oleifera* rootstocks (GW1 and GW2) under P0 and P1 conditions

Principal component analysis (PCA) of 23 integrated traits, spanning growth, biomass, and nutrient parameters, clearly distinguished the two rootstocks (Fig. 7 & Table 3). Under P limited condition, GW1 formed a unique cluster characterized by enhanced root morphological plasticity and balanced shoot-root biomass partitioning. The first two principal components collectively explained 67.3% of the total variance (PC1: 41.6%, PC2: 25.7%). These findings highlight GW1’s rootstock dual capacity for improved P acquisition and efficient P utilization, underpinning its superior adaptive strategy in low-P environments through coordinated whole-plant nutrient management.

**Table 3:** Comprehensive indicator coefficient and contribution rate

	Drought indicator	Component	
		PC1	PC2
Eigenvector	height	0.26788	-0.04679
	diameter	0.16255	-0.33151
	fine root volume	0.23892	0.00748
	small-diameter root volume	0.17462	-0.0791
	thick root volume	0.27723	0.16098
	Root dry weight	0.30687	-0.04729
	shoot dry weight	0.14239	0.31509
	total dry weight	0.28926	0.14584
	root-to-shoot ratio	0.20835	-0.24326
	root P concentration	-3.39E-04	-0.26899
	shoot P concentration	-0.1835	0.05663
	root P uptake efficiency	0.28211	-0.14468
	shoot P acquisition efficiency	0.05769	0.32904
	plant P acquisition efficiency	0.28017	0.03435
	root P utilization efficiency	0.02092	0.25912
	shoot P utilization efficiency	0.10845	-0.37096
	plant P utilization efficiency	0.04723	0.26157
	plant total N content	-0.23853	0.11478
	plant N acquisition efficiency	0.21576	0.21726
	plant N utilization efficiency	0.2262	-0.11964
	plant total K content	-0.12315	0.20187
	plant K acquisition efficiency	0.23526	0.25519
	plant K utilization efficiency	-0.24743	-0.11849
Eigenvalue		9.56	5.9
Contribution rate/%		41.56	25.65
Cumulative contribution rate/%		41.56	67.21

### Gene expression patterns of two rootstocks under P limitation

**Fig. 8:**
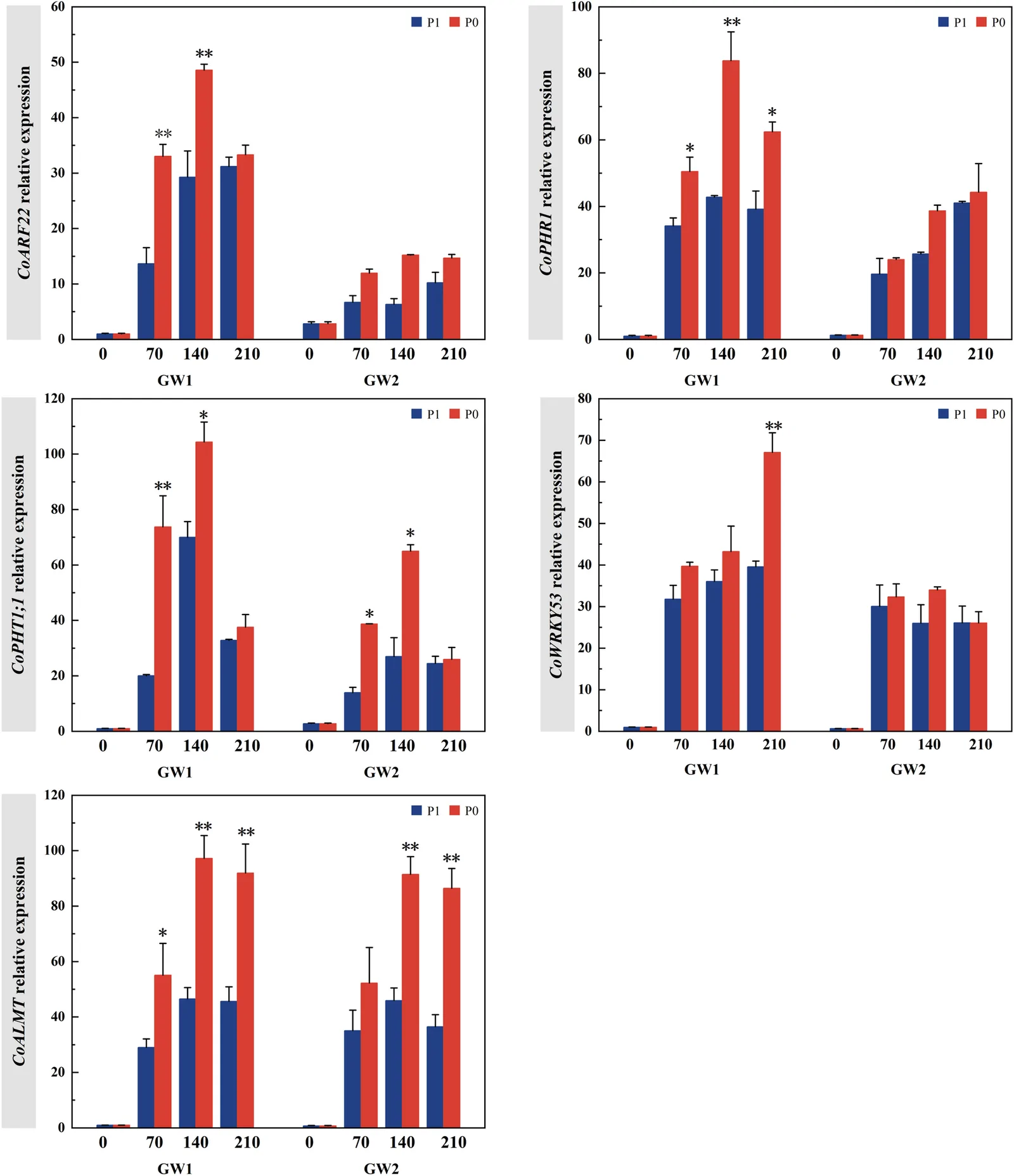
Differential expression of related genes in the roots of two *C. oleifera* rootstocks (GW1 and GW2) under P0 and P1. * and ** indicated significant differences at the *P* ≤ 0.05 and *P* ≤ 0.01 levels, respectively; absence of notation indicated no significant difference (*N* = 3)

Key P-responsive genes exhibited distinct genotype-specific regulation patterns (Fig. 8). The auxin response factor *CoARF22* and P starvation regulator *CoPHR1* displayed opposing expression profiles under P limited condition: *CoARF22* was significantly induced in GW1 rootstock, whereas GW2 rootstock showed only marginal (non-significant) increases compared to P-sufficient conditions. The P transporter gene *CoPHT1;1* was strongly upregulated in both rootstocks under P limitation, with GW1 rootstock demonstrating markedly greater induction than GW2 rootstock during the 70–140 day treatment period. The transcription factor *CoWRKY53* underwent dramatic upregulation exclusively in GW1 after 210 days of low P stress, while GW2 exhibited no detectable change. Notably, the malate transporter gene *CoALMT* revealed the most robust induction among all tested genes in both genotypes under low-P conditions, with GW1 rootstock displaying significantly higher transcriptional activity than GW2 rootstock specifically during early stress (70 days).

P-responsive genes demonstrated genotype-specific regulation (Fig. 8). The auxin response factor *CoARF22* and P starvation regulator *CoPHR1* exhibited contrasting expression patterns under P limited condition, with GW1 showing significant induction while GW2 displayed only marginally higher (non-significant) expression compared to P-sufficient conditions. The P transporter gene *CoPHT1;1* exhibited significant induction in both rootstocks under P limited condition, with GW1 rootstock demonstrating markedly stronger upregulation compared to GW2 rootstock during the 70 to 140 day treatment period. The transcription factor *CoWRKY53* showed substantially upregulation in GW1 rootstock after 210 days of P limited treatment, while expression levels in GW2 rootstock remained unchanged. The malate transporter gene *CoALMT* revealed the strongest induction among all tested genes in both rootstocks under low P stress, with GW1 rootstock showing significantly higher transcriptional activity than GW2 specifically during the early stress phase (70 days).

## Discussion

In grafting systems, stress-tolerant rootstocks enhance whole-plant resilience while retaining scion traits [28]. While research in other woody crops has shown how rootstocks reshape performance under P limitation [29–31], comparable insight for C. oleifera remained scared. Our findings demonstrated that rootstock genotype was a primary regulator of low-P tolerance, with the GW1 combination outperforming GW2 in biomass accumulation and nutrient acquisition, highlighting the value of rootstock selection for sustainable production.

While plant P acquisition is primarily determined by P availability in the soil solution, the root foraging capacity of efficient genotypes plays a crucial role in scavenging P from depletion zones by prioritizing carbon allocation to fine roots [32–34]. In this study, GW1 effectively reconstructed its root architecture, characterized by significant increases in fine-root volume and P accumulation efficiency, ensuring efficient soil exploration (Table 3). This morphological adaptation was underpinned by a strategic shift in biomass partitioning, as GW1 maintained a significantly higher root-to-shoot ratio and total root dry mass (4.12-fold higher than GW2) under low P stress (Fig. 2). Such a prioritized investment in below-ground organs reflects a proactive carbon-allocation strategy that maximizes the return on nutrient acquisition. In contrast, the increased root surface area in GW2 appeared to be a compensatory but inefficient response. Its proliferation was offset by a substantial loss in fine-root projection area, indicating a failure in spatial foraging. This morphological superiority in GW1 directly translates into physiological robustness. Under low P stress, GW1 maintained significantly higher P accumulation across all measured organs (roots, stems, and leaves) than GW2 (Fig. 5), augmented by a systemic optimization of P utilization efficiency. This robust nutrient status supported the stable basal diameter of GW1 and aligns with its more vigorous appearance compared to GW2 (Fig. 3). Similar strategies have been observed in Malus prunifolia and Chistock #1 apple rootstocks, where more vigorous root systems are essential for enhancing P acquisition [35]. Consequently, GW1 employs a more functionally coordinated root strategy than GW2, enabling superior P uptake and achieving a robust, high-efficiency adaptation to low-P conditions.

Internal P reallocation is essential for weathering chronic P shortage [36]. In this study, GW1 demonstrated superior adaptive plasticity by maintaining steady leaf P levels while prioritizing strategic root expansion, reflecting a highly efficient trade-off between above-ground carbon fixation and below-ground nutrient acquisition. In sharp contrast, GW2 leaves exhibited a marked, yet misleading, increase (34.3%) in P concentration under limitation (Fig. 4). This phenomenon represents a “concentration effect” driven by severely stunted growth rather than enhanced uptake. Coupled with a significant decline in leaf PUE (Fig. 5), this high internal P concentration in GW2 underscores a critical bottleneck in P utilization, leading to systemic metabolic disruptions.

The physiological robustness of GW1 was further evidenced by its ability to maintain superior protein and potassium (K) homeostasis under stress. Notably, while low P stress often impairs energy-dependent K absorption [37], GW1 exhibited substantially increased K concentrations in shoots and leaves, suggesting an exceptionally efficient root-to-shoot translocation mechanism that safeguards cellular functions. Furthermore, the stable soluble protein content (SPC) in GW1, compared to the significant decline observed in GW2, underscores its robust metabolic adaptive mechanism (Fig. 6). The observed lag in physiological response compared to earlier hydroponic studies likely stems from the nutrient buffering capacity of soil [38], which afforded GW1 sufficient time for gradual physiological adaptation and proactive root-mediated nutrient exploration [39].

Acid phosphatase (ACP) plays a dual role in plant P homeostasis: intracellular ACP facilitates internal P recycling, while extracellular ACP—secreted by the roots—is essential for mineralizing soil organic P [38, 40]. In this study, total root ACP activity remained stable during the early stress phase, contrasting with findings in certain annual crops [41]. This lag may be attributed to the slow-growing nature of woody species and their adaptation to acidic habitats, where plants may possess higher constitutive tolerance to transient low-P availability [40]. However, prolonged low P stress (140–210 d) significantly enhanced extracellular ACP activity in GW1. Given that organic P is a prevalent fraction in acidic red soils, this late-stage induction suggests a strengthened physiological potential for both internal P mobilization and external organic P utilization in the efficient genotype [38]. It is important to note, however, that the induction of ACP represents a responsive strategy to low P availability rather than a direct indicator of increased soil P supply, as the actual effectiveness of these enzymes in the rhizosphere depends on various soil physicochemical factors [42].

Antioxidant enzymes (SOD, CAT, and POD) are critical for mitigating oxidative damage under P low P stress. While P stress often reduces these levels in certain crops [9, 33], our results showed a significant upregulation of POD activity in both C. oleifera rootstocks (Fig. 6). Notably, the efficient genotype GW1 exhibited a more persistent induction of POD compared to GW2, a pattern also observed in other acid-adapted woody species [37]. This robust enzymatic response in GW1 was coupled with significantly lower MDA accumulation at the final harvest (210 days). Notably, GW1 maintained consistently lower MDA levels across both P gradients compared to GW2. This suggests that GW1 possesses an inherent (constitutive) physiological robustness that prevents excessive lipid peroxidation, thereby maintaining membrane integrity and ROS homeostasis during prolonged P stress [43]. These findings underscore that the synergy between persistent antioxidant induction and mitigated membrane damage is a key determinant of long-term P-stress resilience in *C. oleifera*.

Building on prior molecular studies of *C. oleifera* [10, 22], five hub genes were analyzed to uncover the regulatory network governing rootstock P-adaptation. While expression patterns were generally conserved, significant rootstock-type divergence emerged under low P stress. In the efficient rootstock GW1, *CoARF22*,* CoPHR1*, and *CoPHT1;1* were all markedly up-regulated, whereas in the sensitive GW2, *CoARF22* and *CoPHR1* remained comparable to controls. Our results suggest that GW1 prioritizes this pathway that the elevation of *CoARF22* enhances the transcription of the master regulator *CoPHR1*, which in turn activates the downstream transporter *CoPHT1;1* (Fig. 7). This molecular cascade likely drives the observed fine-root proliferation and increased root-to-shoot ratio, representing a proactive adaptive strategy that successfully maintains P homeostasis and supports overall growth despite the low-P soil environment. Furthermore, *CoWRKY53* and *CoALMT* in GW1 were robustly induced earlier (peaking at 70 d) than in GW2 (140 d). This proactive induction prior to the growth peak suggests that GW1 possesses a more sensitive perception mechanism, potentially triggering transcriptional priming for organic acid exudation to meet high developmental P demands. Additionally, the involvement of *CoWRKY53* in drought and cold stress implies that GW1 may possess broader environmental resilience [15]. However, as direct flux of organic acids was not quantified and gene functions remain to be validated in this species, future multi-omics and metabolic profiling are essential to fully resolve these spatiotemporal P-starvation responses.

## Conclusions

In summary, GW1 is a superior P-efficient rootstock that maintains metabolic stability under low P stress through proactive root reconstruction and enhanced P mobilization. Practically, using GW1 in the highly-weathered red soils of Southern China can reduce chemical fertilizer reliance and production costs. Future research should focus on the long-term field performance of these grafted combinations and the synergistic interactions between rootstocks and arbuscular mycorrhizal fungi (AMF) to further optimize nutrient acquisition in marginal lands.

## Data Availability

All data generated or analysed during this study are included in this published article.

## Abbreviations

P: Phosphorus
PHTs: Phosphate transporters
ALMT: Aluminum-activated malate transporters
POD: Peroxidase
CAT: Catalase
SOD: Superoxide dismutase
ACP: Acid phosphatase
MDA: Malondialdehyde
SPC: Soluble protein content
GW1: Ganwu 1
GW2: Ganwu 2
